# Radiogenomics: a key component of precision cancer medicine

**DOI:** 10.1038/s41416-023-02317-8

**Published:** 2023-07-06

**Authors:** Zaoqu Liu, Tian Duan, Yuyuan Zhang, Siyuan Weng, Hui Xu, Yuqing Ren, Zhenyu Zhang, Xinwei Han

**Affiliations:** 1 Department of Interventional Radiology, The First Affiliated Hospital of Zhengzhou University, 450052 Zhengzhou, Henan China; 2 Interventional Institute of Zhengzhou University, 450052 Zhengzhou, Henan China; 3 Interventional Treatment and Clinical Research Center of Henan Province, 450052 Zhengzhou, Henan China; 4 Department of Respiratory and Critical Care Medicine, The First Affiliated Hospital of Zhengzhou University, 450052 Zhengzhou, Henan China; 5 Department of Neurosurgery, The First Affiliated Hospital of Zhengzhou University, 450052 Zhengzhou, Henan China

**Keywords:** Cancer genomics, Cancer imaging

## Abstract

Radiogenomics, focusing on the relationship between genomics and imaging phenotypes, has been widely applied to address tumour heterogeneity and predict immune responsiveness and progression. It is an inevitable consequence of current trends in precision medicine, as radiogenomics costs less than traditional genetic sequencing and provides access to whole-tumour information rather than limited biopsy specimens. By providing voxel-by-voxel genetic information, radiogenomics can allow tailored therapy targeting a complete, heterogeneous tumour or set of tumours. In addition to quantifying lesion characteristics, radiogenomics can also be used to distinguish benign from malignant entities, as well as patient characteristics, to better stratify patients according to disease risk, thereby enabling more precise imaging and screening. Here, we have characterised the radiogenomic application in precision medicine using a multi-omic approach. we outline the main applications of radiogenomics in diagnosis, treatment planning and evaluations in the field of oncology with the aim of developing quantitative and personalised medicine. Finally, we discuss the challenges in the field of radiogenomics and the scope and clinical applicability of these methods.

## Introduction

Since the genomic revolution in the early 1990s, cancer research has focused on investigating the fundamental causes of diseases at the genetic level to enable precision treatments. Following the completion of the Human Genome Project, genomics has evolved to a more functional level, concentrating on the expression profiles and roles of genes and proteins. Our understanding of cancer genetics has changed the way we think about and treat the disease. A vast number of samples have been used to provide genome-wide, transcriptome, epigenomic, and proteomic data in many cancer types, such as the Cancer Genome Atlas (TCGA) project [1, 2]. Nevertheless, traditional means of genetic analysis rely on invasive biopsy sampling or post-operative pathological tissues to perform the procedure, which carries certain risks and potential complications, therefore cannot be applied to every cancer patient. Due to intra- or intertumoral heterogeneity [3], tissue biopsies may not accurately detect important genetic alterations. Samples are often derived from a small fraction of heterogeneous lesions and may not accurately represent the anatomical, functional, and physiological characteristics of the lesion [4–6]. More importantly, it is impossible to obtain tissue multiple times throughout the treatment to examine the response. Thus, integration of genomic or proteomic profiling into general clinical practice remains difficult [7].

Medical imaging is a crucial technology in medical science and clinical practice [8]. Whereas, the role of medical imaging is rapidly evolving from a major diagnostic tool to a predominant role in the circumstances of personalised precision medicine [9]. Conventional imaging evaluation of tumours depends on qualitative features such as tumour density, enhancement patterns, regularity of tumour margins, intratumor cells and acellular components, the anatomical relationship to surrounding structures, and anatomical changes [10–13]. In contrast, radiomics, a fast-developing area, permits the digital decoding of radiographic pictures into quantitative information gained from the four-channel images, such as intensity, texture, shape and size metrics [14, 15]. Radiomics is a new technology introduced in 2012 [16], focusing on extracting quantitative image features from images through high-throughput algorithms and then filtering, clustering, and analysing these data to identify and predict tumour heterogeneity [17–19]. Despite advances in transforming digital medical images with various relevant data to improve decision-making support, critical barriers exist in identifying biological interpretations of radiomic features [20, 21].

As we enter the next era of precision medicine and big data, many experts have put forward the concept of “Radiogenomics”. Radiogenomics focuses on developing multi-scale connections between medical imaging and genomic data. It can be considered as a combination of radiomics and genomics that addresses the above shortcomings [22]. Over the past decade, it has grown tremendously and has shown great potential to develop non-invasive prognostic and diagnostic approaches to identify biomarkers for therapy, particularly for cancer, by linking quantitative imaging features of tumour phenotypes to genomic signatures [23]. With the improved molecular characterisation of various cancer types and advances in texture analysis and machine learning, cancer diagnostics are poised to move into personalised medicine with radiogenomics [24].

Although several specialists have achieved significant advances in image genomics research, the future development of imaginary genomics confronts massive challenges. The textural characteristics, resolution, and imaging parameters of imaging equipment must be increased further. As the volume of clinical data grows, so will the need for computers and data exchange [25].

Despite the aforementioned limitations, radiogenomics, a field based on advances in computational analysis of medical imaging and bioinformatics, can help overcome several difficulties in this field by contributing to quantifying tumour characteristics, detecting early relapse after treatment, and driving precision medicine forward. This review article highlights the present state of radiogenomics research in tumour characterisation, addresses several of its limitations, promises, and projects its future directions.

### An overview of radiogenomic workflows

**Fig. 1:**
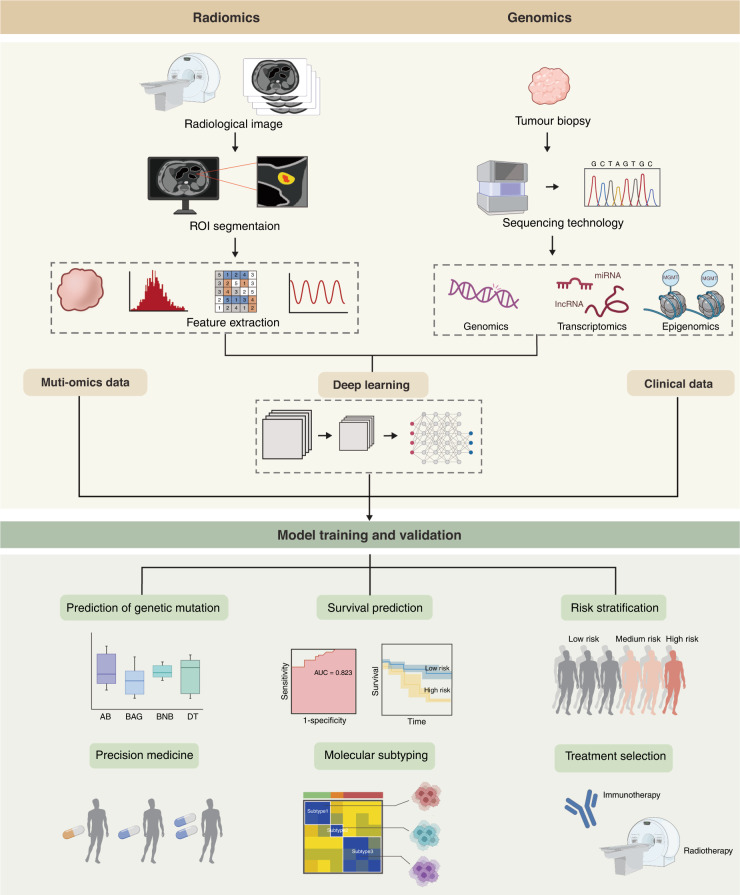
A schematic representation of the integration of radiomics with clinical data, genomic data, and multi-omics data to construct extremely accurate predictive models. The diagram depicts a general radiogenomic study procedure. The initial step involves data collection (clinical information, imaging, and genomic data). The datasets are then standardised and subjected to an integrative analysis to describe each radiomic characteristic and discover unique molecular functions.

The core idea of radiogenomic is to fuse genomic data reflecting molecular-level activities with imaging data reflecting quantitative disease phenotypes to establish the links between genomic and image features, to gain insight into the genetic background and development of diseases based on the analysis and distillation of genomic and imaging information. A detailed introduction to those tools and methods is given below. In 2012, Lambin et al. [1] formally introduced the concept of imaging genomics, which is translating medical images into a large amount of feature information through automated and high-throughput feature extraction methods to explore the biological nature of the images and provide clinical decision support. The radiogenomic workflow consists of the following steps [2]: (i) the image collection is similar to “case enrollment” in clinical trials. A variety of image collection pathways exist, including radiography, ultrasound, magnetic resonance imaging (MRI), and positron emission tomography (PET)/CT; (ii) image segmentation, includes manual segmentation, semi-automatic segmentation, and fully automatic segmentation; (iii) image feature extraction and identification, i.e., extracting high-dimensional feature data to quantitatively describe the properties of regions of interest (ROI), which is the core part of image histology [1]; (iv) feature selection and model building; (v) clinical applications, where image histology is most applied, include tumour classification, tumour staging and prognosis prediction (Fig. 1).

### Several multiple omics major branches of radiomicsgenomics

The recent development of radiogenomics technologies has brought paradigm shifts to investigating radiomics clinical application from a multi-omic perspective. Fathi et al. [26] fuse multiple omics data, namely clinical information, MGMT methylation, radiomics and genetics, to accurately model clinical outcome in patients with newly diagnosed GBM. This research has characterised the radiogenomic application in precision medicine using a multi-omic approach for accurate stratification of risk groups. Accordingly, in this review, we break radiogenomics down into three primary components. We combed the PubMed and Web of Science databases for relevant papers published between 2017 and 2022 with impact factors (IF) over 5, as shown in Table 1.

**Table 1:** Description of pan-cancer radiomic studies focused on genomics.

Cancer type	Genomic feature	Imaging modality	Statistics and modelling	Patients	Result	References
Gliomas						
	IDH1 mutation	MRI	Multivariate RF classifier model	Training, *n* = 100 Validation, *n* = 100	The combined model produced a maximum AUC of 78.24%.	[3]
	IDH1 mutation	MRI	the SVM-based recursive feature elimination (SVM-RFE) algorithm		The AUC of the predicted IDH1( + ) STATUS is 94.74%.	[4]
	IDH1 mutation	MRI		Training, *n* = 60 Validation, *n* = 20	The DWI-trained XGBoost model performed best, which achieved ROC on the test set with an area under the curve (AUC) of 0.97.	[5]
	IDH mutation	MRI	Convolutional Neural Networks	*n* = 214	T2-net demonstrated a mean cross-validation accuracy of 97.14% ± 0.04 in predicting IDH mutation status.	[6]
	IDH mutation	MRI	Convolutional Neural Networks	Training, *n* = 727 Validation, *n* = 129	The hybrid model achieved accuracies of 93.8%, 87.9% and 78.8%.	[7]
	ATRX mutation	MRI	Linear SVM model and random forest model	95	94.0%	[8]
	ATRX mutation	18F-FET/PET and MRI	Support vector machines and random forest models	42	The five fold cross-validated area under the curve in predicting the ATRX mutation was 85.1%.	[9]
	ATRX mutation	MRI	the Elastic Net regression model	111	The radiomics nomogram identified LrGG patients for ATRX loss (C-index: training sets = 0.863, validation sets = 0.840).	[10]
	MGMT methylation	MRI	Multiple logistic regression model	Training, *n* = 105 Validation, *n* = 31	The fusion radiomics signature exhibited supreme power for predicting MGMT promoter methylation, with an AUC of 0.925 in the training cohort and 0.902 in the validation cohort.	[11]
	MGMT methylation	18F-DOPA-PET	*Random forest* models	Training, *n* = 59 Validation, *n* = 10	Achieved 80% ± 10% accuracy for a 95% confidence level in predicting MGMT status.	[12]
Glioblastomas						
	MGMT methylation	MRI	random forest models	Training, *n* = 130 Validation, *n* = 60	Radiomics model built from multiregional and multiparameter MRI may serve as a potential imaging biomarker for pre-treatment prediction of MGMT methylation in GBM.	[13]
	MGMT methylation	MRI	Multivariate Cox model	Training, *n* = 120 Validation, *n* = 61	Radiological characteristics together with MGMT status were the only parameters with independent significance in the multivariate analysis (*P* ≤ 0.01).	[14]
	MGMT methylation	MRI	multivariable Cox-regression model	Training, *n* = 142 Validation, *n* = 46	The predictive model performed significantly in the external validation of MGMT methylation (AUC 0.667, 95% CI 0.522–0.82).	[15]
NSCLC						
	EGFR mutation	^18^F-FDG-PET and CT	CS model/ multivariable logistic regression analyses	Training, *n* = 429 Validation, *n* = 187	Deep-learning score (EGFR-DLS) is significantly and positively associated with longer progression-free survival (PFS).	[16]
	EGFR mutation	MRI	Gradient boosting classifier model	Patients=110	Data support the use of radiological scores based on MR imaging of NSCLC brain metastases as a non-invasive biomarker of survival.	[17]
	KRAS mutation	CT	LASSO regression model	Training, *n* = 145 Validation, *n* = 101	This diagnostic/prognostic study examined a CT-based DL approach to predict the efficacy of EGFR-TKI therapy in patients.	[18]
	KRAS mutation	CT	LASSO regression model	134	The AUCs for the combined models used to identify KRAS and TP53 mutations were 0.81, and 0.84, respectively.	[19]
	KRAS mutation	PET and CT	Radiomics score (RS) models	Training, *n* = 180 Validation, *n* = 78	the PET/CT radiomics score model exhibited a higher AUC for predicting KRAS mutations (0.83).	[20]
	ALK rearrangements	CT	Generalised boosted regression model (GBM)	Training, *n* = 84	The average accuracy of the model calculated on the independent nested validation set was 0.81.	[21]
	ALK rearrangements	CT	LASSO regression model	Training, *n* = 268 Validation, *n* = 67	The addition of conventional CT features enhanced the validation performance of the radiomic model in the primary cohort (AUC = 0.83–0.88).	[22]
	ALK rearrangements	PET and CT	LASSO logistic regression	Training, *n* = 368 Validation, *n* = 158	This combined model PET/CT clinical model has a significant advantage to predict the ALK mutation status in the training group (AUC = 0.87).	[23]
Colorectal cancer						
	KRAS mutation	CT	RELIEF and support vector machine methods	Training, *n* = 61 Validation, *n* = 56	The AUC, sensitivity, and specificity for predicting KRAS/NRAS/BRAF mutations were 0.86.	[24]
	KRAS mutation	MRI	SVM classifiers	Training, *n* = 213 Validation, *n* = 177	The proposed T2WI-based radiomics signature has a moderate performance to predict KRAS status.	[25]
	KRAS mutation	CECT	Artificial neural network method (ANN)	Training, *n* = 93 Validation, *n* = 66	The combined score could distinguish between wild-type and mutant patients with an AUC of 0.95 in the primary cohort.	[116]
Clear cell renal cell carcinoma						
	BAPI mutation	CT	Random forest model	Patients=54	The AUC of the random forest model for predicting the mutation status of BAP1 was 0.77.	[117]
	VHL mutation	CT	Random forest model	Training, *n* = 170 Validation, *n* = 85	The model with eight all-relevant features achieved an AUC of 0.949 in the validation cohort.	[118]
	VHL mutation	CT	Random forest model	Training, *n* = 207 Validation, *n* = 175	Using radiomics features, the random forest algorithm showed a good capacity to identify the mutations *VHL* (AUC = 0.971).	[119]

#### Using radiogenomic models to predict the spectrum of mutated genes

Identifying gene mutations is crucial for cancer diagnosis, treatment selection and monitoring of treatment effectiveness. The most common method for detecting these gene mutations is tissue biopsy, which is invasive, expensive, and time-consuming and thus unavailable for all patients. Concurrently, radiomics refers to the automated extraction and analysis of large quantities of advanced quantitative imaging features obtained from different imaging modalities using standard radiological scans. The field of radiogenomics has recently emerged that integrates radiomics and genomics, possibly facilitating precision medicine. Hence, further study of radiomic application in genomics is warranted to help guide more effective cancer detection. In this part, we briefly review our present knowledge of the role of radiomics in predicting gene mutations in brain, lung, colorectal, breast, and kidney tumours. Most studies focused on gene mutations including single-nucleotide substitutions and insertions/deletions (indels).

Single-nucleotide polymorphism (SNP) is the most widely studied genomic variant [27, 28]. SNPs are single-base pair alterations that can affect coding and noncoding DNA and protein expression, while GWAS is a molecular genetic study that uses millions of SNPs in the genome for genetic analysis [29, 30]. GWAS uses millions of single-nucleotide polymorphisms in the genome as molecular genetic biomarkers to compare and find genetic variants affecting complex traits [31]. Seibold et al. [32] used radiogenomics to evaluate the ten most important SNPs in four genes at the replication stage in 1883 breast cancer patients and then validated them in 753 breast cancer patients, finding that rs2682585 in XRCC1 was strongly associated with late skin toxicity and overall toxic response. Similarly, Kerns et al. [31] discovered that AGT, COG2, CAPN9, ARV1, AL512328.1 and LOC101927604 might be related to progressive radiotoxic haematuria after prostate cancer radiation.

Since specific therapies are available for genomic subgroups of non-small cell lung cancer (NSCLC), genotyping is crucial for directing therapy [33]. Radiogenomics could potentially provide an important technological tool for the rapid non-invasive genotyping of tumours. Epidermal growth factor receptor (EGFR) mutations and anaplastic lymphoma kinase (ALK) rearrangements are crucial biological indicators for treating NSCLC patients with tyrosine kinase inhibitors (TKIs) [34, 35]. Lv et al. [36] confirmed that low pSUVmax is integrated with mutant EGFR status and could be associated with other clinical factors to improve the discriminability of the EGFR mutation status in several NSCLC patients whose EGFR testing is unavailable. A total of 849 NSCLC patients with EGFR or ALK alterations were enrolled in this retrospective study. Zhang et al. [37] used the LIFEx package to extract 47 PET and 45 CT imaging features to filter ten imaging histology features, which were combined with clinical variables to create a predictive model that showed high predictive power for EGFR. Similarly, Zhang et al. [38] predicted EGFR mutations in NSCLC from 485 quantitative texture features from CT images of training group patients, revealing that the prediction model based on imaging histological features had excellent performance. Nair et al. [39], on the other hand, developed an imaging genomics model that identified EGFR mutations using CT and 18F-FDG-PET-CT images. With an AUC of 0.8, the FDG-PET-based model could differentiate between wild-type and mutant EGFR. Altogether, FGD-PET and CT imaging have robust performance in identifying EGFR mutations and ALK rearrangements in NSCLC tumours.

The increased incidence of colorectal cancer (CRC) with the operator increase in morbidity and mortality, poses an enormous therapeutic challenge [40–42]. In recent years, widespread interest has been attracted in imaging genomics studies regarding KRAS mutation status in CRC patients. Shin et al. [43] observed that polyp morphology, increased axial length, increased axial-to-longitudinal ratio, and N2 lymph node status was related to KRAS mutations. Subsequently, a study by Lubner et al. [44] examined 77 CRC liver metastases and found that texture parameters correlated with tumour grade, serum carcinoembryonic antigen (CEA), and KRAS mutation status. Yang et al. [24] modelled and analysed the relationship between gene mutations and clinical background, tumour stage, and histological differentiation in 117 CRC patients. The findings implied that imaging histological features correlated with KRAS/NRAS/BRAF mutations, and CT may have contributed to CRC tumour genotypes and further facilitate precise therapy.

The above studies have shown that conventional imaging features and imaging histological features have good predictive value for gene mutations in several cancers. Although cannot replace puncture biopsy with the advantages of being non-invasive and reproducible, they may assist in clinical treatment decisions. Nonetheless, the clinical applications may be limited due to numerous yet-to-be-discovered radiosensitive SNPs.

#### Radiomic applications in transcriptomics

Understanding the numerous roles that each gene may play requires knowledge of transcriptomics, which is the pattern of gene expression at the level of genetic transcription in a particular organism or under particular conditions in particular cells. Transcriptomics can be used in medicine to better understand the variations in gene expression between healthy people and sicks. Researchers are given hints into how variations in gene expression can affect the progression of the disease by researching which genes are switched on and which are shut off, and in what groups of people. Therefore, further research on radiomic applications in transcriptomics is necessary to help direct additional accurate cancer detection. The function of radiomics in transcriptional biomarkers in tumours of the brain and kidney is briefly reviewed in this section.

The clinical significance of some prevalent mutations in ccRCC remains unknown, limiting their utility as clinical biomarkers. Transcriptional biomarkers, on the other hand, are superior methods for categorising ccRCC into clinically meaningful molecular subgroups. Using next-generation RNA-sequencing data, Brooks et al. [45] established a 34-gene expression signature for classifying localised ccRCC into high-risk and low-risk categories. In addition, Jamshidi et al. [46] performed the GSEA algorithm to identify diverse oncogenic pathways using MR imaging features. Correlations between 34-gene loci were discovered, which revealed concordant variations in gene dosage and mRNA expression, yielding an MR imaging, mRNA, and CNV radiogenomic association map for GBM. Another study probed transcriptomics associating molecular features with 18F-fluorocholine PET/CT imaging phenotypes and its potential relationship to survival in hepatocellular carcinoma to provide a pathobiological framework [47]. Similarly, using a radiogenomic association map links MR image phenotypes to global gene expression patterns in breast cancer, Yamamoto et al. [48] defined associations between specific MR image phenotypes and gene sets of interest to understanding the underlying molecular biology of breast cancers. They investigated the relationship between 47 elevated MR image features and long noncoding RNAs and found that MR image edge enhancement has been demonstrated related to eight lncRNAs, including HOX transcriptional antisense RNA (HOTAIR).

#### The combination of radiomics and epigenetics

Gaining insight into how epigenetic alterations drive cancer formation is an area of intensive interest in cancer research. As opposed to genetic mutations, which in the case of cancer are essentially irreversible, epigenetic modifications are reversible and thus represent attractive targets for intervention [49]. Cancer is initiated and developed by epigenetic mechanisms, and epigenetic diversity encourages dynamic gene expression patterns that aid tumour evolution and adaption. [50–52]. Radiogenomics give new approaches to the important relationships between epigenetic traits and cancer clinicopathological characteristics, highlighting the potential for epigenetic marks to function as biomarkers in the context of precision medicine.

To investigate the relationships between CT imaging features, RUNX3 methylation levels, and survival in clear cell renal cell carcinoma (ccRCC), Cen et al. [53] constructed a model of renal clear cell carcinoma and discovered that the presence of indistinct tumour margins, left-sided tumours, and intra-tumour vessels significantly predicted the elevation of RUNX3 methylation levels. Furthermore, Kanas et al. [54] demonstrated an association between standard preoperative MRI variables and MGMT methylation status in glioblastoma.

Overall, it has become clear that a better understanding of epigenetic mechanisms and the interplay among epigenetic omics and radiomics may provide new insights for developing radiogenomics strategies.

### Clinical applications

Since the beginning of this decade, radiogenomics research has made alarming progress, highlighting the potential of the field to significantly advance clinical care. Given that radiogenomics is still in its infancy, the full potential of clinical translation is yet to be realised. Nevertheless, several studies have shown early promise for clinical applications. We combed the PubMed and Web of Science databases for relevant papers published between 2017 and 2022 with impact factors (IF) over 5, as shown in Table 2.

**Table 2:** Description of pan-cancer radiogenomic studies focused on clinical applications.

Cancer type	Study objective/genomic feature	Radiomic feature	Imaging modality	Machine-learning algorithm	Patients	Study type	Author
Breast cancer							
	Oncotype Dx RS	Handcraft	MRI	None	43	Retrospective study	Thakur et al. [120]
	Oncotype Dx RS	Deep	Mammographic and MR	Linear regression models	556	Retrospective study	Woodard et al. [98]
	Oncotype Dx RS	Handcraft	MRI	Convolutional neural network (CNN) algorithm	134	Retrospective study	Ha et al. [121]
	Oncotype Dx RS	Deep learning	MRI	None	382	Retrospective study	Li et al. [122]
	Predicting molecular subtypes	Deep	MRI	Convolutional neural network (CNN) algorithm	213	Retrospective study	Ha et al. [123]
	Molecular subtypes	Deep	MRI	None	132	Prospective study	Iima et al. [124]
	Molecular subtypes	Handcraft	MRI	Linear regression	306	Prospective study	Tsai et al. [125]
	Molecular subtypes	Deep	MRI	Convolutional neural network (CNN) algorithm	244	Retrospective study	Zhang et al. [126]
Clear cell renal cell carcinoma							
	Molecular subtypes	Handcraft	PET and MRI	multivariate logistic regression analysis	77	Retrospective study	Wang et al. [127]
	Molecular subtypes	Handcraft	Enhanced CT	Multivariate logistic regression analysis	131	Retrospective study	Gao et al. [128]
Medulloblastoma							
	Molecular subtypes	Handcraft	MRI	Multivariate logistic regression analysis	111	A combined retrospective and prospective study	Dasgupta et al. [129]
Gliomas							
	Molecular subtypes	Handcraft	MRI	Cross-validation model	110	Retrospective study	Buda et al. [130]
	Molecular subtypes	Handcraft	18F-Fluorocholine PET/CT	ML was not used to build the predictive model	35	Prospective study	Garcia et al. [131]
	Molecular subtypes	Handcraft	MRI	Multivariate logistic regression analysis	272	Retrospective study	Nam et al. [132]
	Molecular subtypes	Handcraft	CT/MRI	Logistic regression analysis	189	Retrospective study	Zhao et al. [133]
	molecular subtypes	Deep	MRI	Convolutional neural network (CNN) algorithm	1016	Retrospective study	Li et al. [134]
Hepatocellular carcinoma							
	Identifying microvascular invasion (MVI)		CT	Multivariate logistic regression analysis	495	Retrospective study	Dasgupta et al. [135]
	Identifying microvascular invasion (MVI)	Handcraft	CT	LASSO logistic regression and logistic regression analysis	145	Retrospective study	He et al. [136]
	Identifying microvascular invasion (MVI)	Handcraft	CT	Logistic regression analysis	185	Retrospective study	Liu et al. 2021 [137]

#### The relationship between imaging phenotypes and molecular phenotypes

Obtaining an accurate molecular phenotype is a prerequisite for targeted therapy. Due to the heterogeneity of malignant tumours, it is difficult to accurately reflect tumour gene mutations in small pieces of tissue obtained by biopsy [55, 56]. Radiogenomics is dedicated to revealing the relationship between imaging features (imaging phenotype) and molecular markers (molecular phenotype) of tumours, thus improving the above situation [57, 58].

The detection of IDH1 mutations holds great diagnostic and prognostic significance for glioma. GBM can be classified as IDH-wild type or IDH-mutant type based on isocitrate dehydrogenase (IDH) status [59]. Most investigations have used MR imaging to predict IDH status, with modest success. Based on clinical factors and MRI multimodal characteristics, the current study applied machine-learning methods to predict IDH genotypes in high-grade gliomas. For instance, Zhang et al. [60] constructed a random forest classifier that used clinical data with multimodal, preoperative imaging features to predict IDH genotypes in high-grade gliomas. Besides, Chang et al. [61] performed the deep-learning technique to non-invasively predict IDH genotype in grade II-IV glioma, capitalising on conventional MR imaging using a multi-institutional data set. The model has the potential to serve as a non-invasive tool that complements invasive tissue sampling, facilitating patient management at an earlier stage of disease and during the follow-up.

**Fig. 2:**
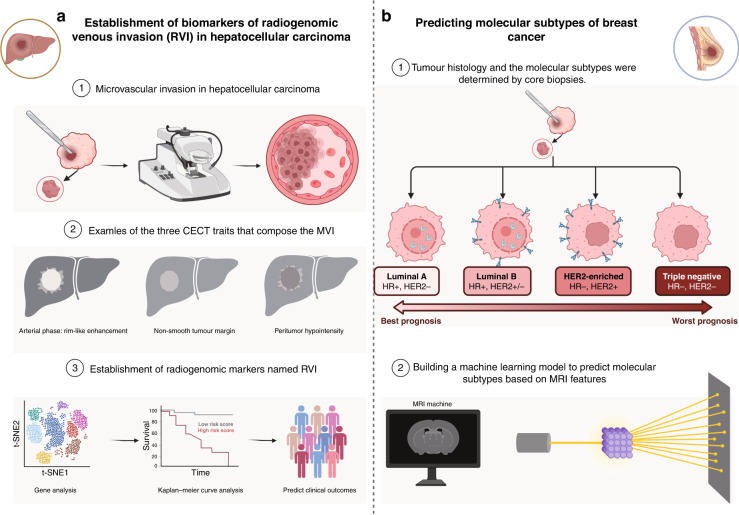
Utilisation of radiogenomics in clinical practice for the treatment of hepatocellular carcinoma and breast cancer. **a** Combining imaging features of hepatocellular carcinoma with genetic modules to build models for predicting prognosis and recurrence. **b** Breast MR imaging radiogenomics allows for the assessment of correlations between imaging features and breast cancer molecular subtypes of luminal A, luminal B, HER2 and triple-negative cancer.

Breast cancer molecular subtypes, including luminal A, luminal B, HER2-enriched, and basal-like (Fig. 2), were suggested firstly by Perou et al. [62] as an approach to explain the differences in therapeutic responses and patient outcomes beyond what could be achieved alone by tumour nuclear grade and size. A considerable amount of subsequent work has validated the clinical relevance of these molecular subtypes and systemic therapeutic decisions for chemotherapy, endocrine therapy, and HER2-targeted therapy partly [63–65]. Mazurowski et al. [66] examined 48 patients from the cancer imaging archive and retrieved 23 DCE-MRI features that revealed a link between luminal B breast cancer and dynamic enhancement features. That is, the luminal B subtype tended to possess a higher ratio of lesion enhancement to background parenchymal enhancement. Leithner et al. [67] found that DWI image characteristics, such as first-order histograms and grayscale covariance matrices, could be more accurate in identifying breast cancer receptor status and molecular subtypes, notably for luminal B and HER2-enriched subtypes. Xie et al. [68] employed multiparametric MR imaging and whole-tumour histogram analysis to distinguish triple-negative breast cancer from other subtypes of breast cancer and demonstrated good accuracy in the differential diagnosis of triple-negative breast cancer from Luminal A and HER2-enriched subtypes. Dilorenzo et al. [69] investigated the value of MRI background parenchymal enhancement (BPE) for the differential diagnosis of different breast cancer subtypes and showed that mild BPE suggested Luminal B or HER2-negative subtypes, while severe BPE suggested triple-negative breast cancer.

New biological insights have led to the recognised classification of medulloblastoma (MB) into four distinct molecular subgroups-sonic hedgehog (SHH), wingless (WNT), group 3, and group 4 [59, 70]. The conventional imaging features extracted from preoperative multiparametric MRI were correlated with molecular subgroups in MB, allowing the construction of subgroup-specific nomograms with variable predictive accuracy. Preoperative multiparametric magnetic resonance imaging based on nomograms can reliably predict molecular subtypes of SHH and group 4 medulloblastoma. Yan et al. [71] developed machine-learning models for predicting molecular subpopulations of MB. The results showed that machine-learning algorithms offered the potential to non-invasively predict molecular subpopulations.

#### Radiogenomics as a tool for assessing the efficacy of oncology treatments and selecting treatment options

Recent years have witnessed unprecedented progress in the research of imaging genomics, and the image characteristics of tumour tissues can be used to precisely predict the response to diverse therapies, including chemotherapy, radiotherapy, targeted therapy and immunotherapy [72–77].

##### Immunotherapy

18F-FDG-PET/CT imaging genomics has been increasingly studied and applied to predict PD-1/PD-L1 expression [78–80]. A recent study found that DLS (deeply learned score) can be served as a substitute for PD-L1 measurement as determined by IHC to guide individual pre-treatment decisions pending in larger prospective trials [72]. Similarly, Dall’Olio et al. [74] showed that total metabolic tumour volume (tMTV) ≥ 75 cm^3^ could be a biomarker of poor prognosis in patients with advanced NSCLC and high PD-L1 expression who were administered with first-line pembrolizumab. The information could be useful in identifying patients who may benefit from the addition of chemotherapy to pembrolizumab.

##### Neoadjuvant systemic therapy

Neoadjuvant systemic therapy (NST) is the standard care for localised and advanced breast cancer, reducing tumour size and increasing opportunities for breast-conserving surgery [81]. However, few breast cancer patients benefit from NST treatment, as some biologically aggressive lesions may not be effectively controlled after several months of NST treatment. Accordingly, it is critical to identify patients who could benefit from NST therapy. Pathological complete response (pCR), closely associated with a long-term favourable prognosis, can be used as an indicator to evaluate the effectiveness of NST treatment. Tsukada et al. [82] predicted whether tumour types would reach pCR after NST completion or not and revealed that the two MRI-derived features (tumour growth direction and contouring rate) were associated with pCR. The research indicated that the tumour growth direction parallel to the Cooper ligament and the rapid contouring rate on pre-treatment multiparametric MRI were predictors of pCR. Kim et al. [77] used recurrence-free survival to evaluate the prognosis of breast cancer and discovered that patients with high entropy (high heterogeneity) at T2WI had significantly lower recurrence-free survival.

##### Radiotherapy

In the peri-radiotherapy period, radiogenomics can provide an integrated model that encompasses both imaging and genetic dimensions to assist clinical decision-making.

Firstly, numerous studies have demonstrated that radiogenomics can predict the adverse effects of cancer radiotherapy and identify genetic markers, thereby facilitating the selection of the best treatment regimen based on genetic factors and other tumour characteristics to maximise treatment outcomes [75, 83–85]. Secondly, radiotherapy techniques nowadays have evolved from two-dimensional radiotherapy to three-dimensional radiotherapy and even four-dimensional radiotherapy techniques; radiotherapy dose distribution has also evolved from point dose to volume dose distribution, etc. These new radiotherapy techniques require a large amount of imaging data as the basis. Radiomics techniques enable detailed and accurate image phenotyping and demonstrate intra-tumoral heterogeneity in a wide range of solid tumours [86, 87]. Thirdly, multi-omics characterisation was extracted and incorporated into the physical model, which contributed to guiding radiotherapeutic physicists to augment reirradiation protocols in targeted therapies.

##### Predict drug response and potential resistance

Radiogenomics can also be used to predict drug response and potential resistance to guide individualised treatment of tumours. As early as 2007, Kuo et al. [88] found a strong correlation between specific imaging presentations on enhanced CT and sensitivity of hepatocellular carcinoma to adriamycin. Precision medicine requires not only the identification of modifiable surveillance and therapeutic targets but also the development of reliable, non-invasive technology for detecting changes in these targets over time. Radiogenomics can give voxel-by-voxel genetic information for a single, heterogeneous tumour or, in the case of metastatic disease, a collection of cancers, supporting the formulation of personalised treatment programs [89].

#### Radiogenomic models as clinical biomarkers to predict prognosis and recurrence

**Fig. 3:**
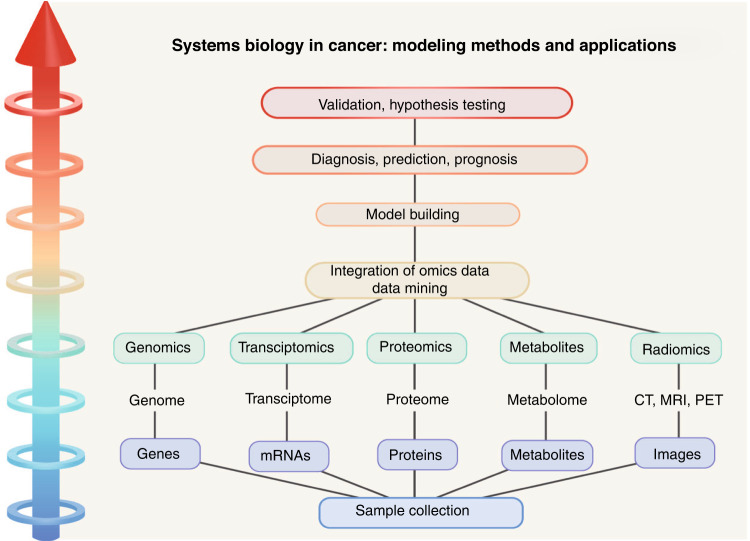
A general hierarchical diagram of the systems biology approaches toward diagnosis and prognosis of cancer. The collection of carcinoma focal for “omics” analysis and the integration of imaging into the omics paradigm enables data mining, model development, and therefore a rise in diagnostic, prognostic and therapeutic prediction accuracy.

The establishment of radiogenomics tags by fusing imaging, genetic and pathological features reveals the link between imaging and patients’ outcomes, which can accelerate the introduction of radiogenomics to clinical applications [90] (Fig. 3).

Developing alternative image biomarkers empowers the ability of clinicians to predict clinically relevant outcomes. Radiogenomic venous invasion (RVI) is a contrast-enhanced computed tomography (CECT) biomarker of MVI (Fig. 2). Banerjee et al. [90] assessed RVI capability and discovered that preoperative CECT is related to poor OS and early disease recurrence. RVI may be effective in identifying individuals who are less likely to gain a durable benefit from surgical treatment.

The application of multigene tests to predict the risk of tumour recurrence has been implemented in clinical practice, such as Oncotype Dx and Prediction analysis for microarrays (PAM50) [91–94]. Oncotype Dx is a recurrence score by evaluating the RNA expression of 21 genes [95]. Ashraf et al. [96, 97] first investigated the association between 21-gene recurrence scores and imaging genomics. Woodard et al. [98] found that breast density was negatively correlated with Oncotype Dx recurrence score (ODxRS), with indistinct mass margins and elongated linear branch calcifications significantly associated with higher ODxRS. In addition to mammographic features, dynamic enhancement features in MRI may also be an imaging marker of breast cancer recurrence risk. Li et al. [99] used multiple genetic tests (MammaPrint, Oncotype DX and PAM50) against computer-derived breast MRI phenotypes, and a significant correlation was identified between imaging histological features, especially tumour size and enhancement texture, and recurrence scores from multiple genetic tests. The studies above improve our understanding of transcriptomic signature and radiogenomics tags and further provide promise for image-based phenotyping in assessing the risk of breast cancer recurrence [100–102].

The first radiogenomic risk scores (RRS) for kidney cancer were created by Jamshidi et al. [103], which consisted of four CT imaging features (up to the presence of tumour necrosis, infiltration of the transitional zone, the presence of discontinuous enhancing margins of the tumour, and the presence of attenuated tumour margins). Of note, independent of disease stage, grade, and other clinical manifestations, RRS was valuable in predicting disease-specific survival. Subsequently, several studies confirmed the reliability of RRS as an indicator of disease-specific survival, and it was negatively correlated with survival [104, 105].

### Challenges in current radiogenomics clinical practice

Due to thousands of quantitative radiomics features being present in the radiological images, in most cases, the deep-learning algorithms automatically extract and select the desired and meaningful deep features rather than hand-crafted traditional methods for conventional radiomics features. Hence, robust deep-learning algorithms for developing reliable models are necessary [106]. These methods can learn from data, automating and enhancing the process of prediction and improving the performance of radiomics-based predictive models. Multiple machine-learning algorithms were assessed for the training of the model in patients with cancer by Jena et al. and Saxena et al. [107, 108]. The authors emphasize the importance of adopting proper machine-learning strategies for every form of cancer. Nonetheless, excessive features may contain redundant or unnecessary data, leading to overfitting. Before being considered as input to machine-learning training, the number of features can be reduced by, for example, doing test–retest analysis on patients or phantoms (to choose the most reliable/repeatable features) and assessing redundancy via correlation measures.

Despite the significant promise of radiogenomics analysis in diverse oncologic applications, the primary limitations of several research involve variability in feature extraction and lack of reproducibility. In a two-phantoms study to identify reproducible and non-redundant radiomic features for computed tomography, Berenguer et al. discovered that only 71 of the 177 radiomic features extracted from CT images were reproducible and that only 10 radiomic features were retained because of redundant information [109]. At the same time, Traverso et al. found that first-order features were more repeatable than shape metrics and textural parameters. Entropy was one of the most stable first-order characteristics [110]. Therefore, Future radiomics investigations could benefit from standardising the imaging methodology in terms of dose administration, acquisition parameters, and the use of reconstruction kernels with lower noise levels.

Thirdly, gene expression and signalling pathways are extremely complex while sequencing is expensive and complicated, which limits large-scale imaging genomics studies. Fourthly, the lack of consistent standards also impacts feature extraction and image correlation analysis. Variations in software and imaging equipment, differences in datasets between and within institutions, and methods of segmenting ROIs, for example, can all impact feature extraction [111].

Lastly, the majority of studies are retrospective with small sample sizes. On the other hand, to evaluate the link between various oncological features and the related genes of interest, the majority of studies relied on single-centre patient cohorts. When there are insufficient samples, the stratification of training, validation, and testing datasets are inadequate, which has a detrimental impact on the model adaption, optimisation, and assessment processes.

## Discussion

Advances in high-throughput imaging technology have spearheaded the brand new generation of “omics” research and the increasing availability of complex data elements obtained from “omics” technology. Imaging plays a critical role in promoting the development of genome-driven signatures and the new domain of radiogenomics. Radiogenomics is an emerging interdisciplinary field that exploits the relationship between medical images and genomic data to identify biological markers that can reflect genetic characteristics. It plays a significant non-invasive role in disease diagnosis, individualised treatment, prognosis prediction and efficacy evaluation.

Despite the technical challenges that lie ahead, we have reason to be optimistic based on the progress that this domain has seen over the past few years. Grand challenges ahead for radiogenomics that we are particularly excited about in the sense that we think they could contribute to accelerating progress across the board. Firstly, prospective, multicenter clinical trials and the generation of huge shared radiogenomics datasets might be used by research teams worldwide to formulate and evaluate innovative radiogenomics strategies. These could be an intriguing method for advancing the quality of radiogenomics studies and facilitating their incorporation into clinical practice. Thus, scientists should be motivated to contribute to existing datasets [112]. To establish a robust database for radiomics imaging of cancer, researchers must have unrestricted access to materials such as gene expression and molecular features and radiomics data.

Secondly, standardising analytical methodologies and image collection techniques are crucial for reproducibility across institutions. The Radiologic Society of North America and the Quantitative Imaging Network (QIN) are establishing consensus standards and digital phantoms to facilitate the clinical application of radiogenomics [112–114]. Thirdly, radiogenomic studies are prone to overfitting and/or selection bias, and the continuous emergence of better algorithms (e.g., deep learning, neural networks) may optimise the data. Visualisation of deep-learning characteristics and prediction models could potentially aid in resolving this issue [113].

Investigators should evaluate radiomics quality relying on homogeneous evaluation criteria and reporting guidelines to enhance the robustness and generalisability of future radiogenomics models [115]. In addition, the transparent reporting of a multivariable prediction model for individual prognosis or diagnosis (TRIPOD) statement could give writers additional instructions for developing or validating prediction models.

## Conclusions

In summary, this review provides an overview of radiogenomic research methods and summarises the current radiogenomic achievements. The focus of this article is to provide insight into the strengths and limitations of radiogenomics as a key component of precision cancer medicine, providing clinicians with valuable information to help guide more effective cancer detection and treatment strategies. The findings indicate that radiogenomics reflects the progression of radiology-pathology from the anatomichistologic level to the genetic level and characterises the interface between biological systems methods and imaging. The ultimate target of radiogenomics is evaluating imaging biomarkers incorporating phenotypic and genotypic metrics in the expectation of forecasting outcomes and stratifying patients for more precise therapeutic management. With the growth of clinical data and improved machine-learning approaches, it will play an increasingly essential role in the objective of non-invasively uncovering relevant features that reflect the potential biological functions most strongly related to clinical outcomes.

## Data Availability

Not applicable.
